# Guard cell starch and malate metabolism facilitate stomatal opening in response to low CO_2_

**DOI:** 10.1111/nph.70639

**Published:** 2025-10-11

**Authors:** Fernanda A. L. Silva‐Alvim, Lucia Piro, Diana Santelia, Michael R. Blatt

**Affiliations:** ^1^ Laboratory of Plant Physiology and Biophysics, Bower Building University of Glasgow Glasgow G12 8QQ UK; ^2^ Institute of Integrative Biology ETH Zürich 8092 Switzerland

**Keywords:** *Arabidopsis thaliana* L, guard cells, low CO_2_, malate, OnGuard modeling, starch metabolism, stomatal opening, sugar transport

## Abstract

- Guard cells adjust turgor to regulate stomatal aperture, integrating ion transport with metabolic adjustments. The effect of reduced CO_2_ on stomatal movement, expected under enhanced photosynthesis, remains largely unexplored.
- We exposed *Arabidopsis thaliana* plants to low CO_2_ before light onset and analyzed guard cell starch turnover, malate (Mal) accumulation, and stomatal conductance in wild‐type and mutant lines.
- Low CO_2_ triggered rapid starch degradation and increased Mal levels in guard cells, accelerating stomatal opening. Mutants defective in starch degradation or hexose uptake showed impaired responses.
- These findings demonstrate that low CO_2_ rewires guard cell metabolism through both internal and external carbon sources, enhancing the responsiveness to light and offering new avenues for stomatal bioengineering.

## Introduction

Stomata are tiny pores on the leaf surface that regulate carbondioxide (CO_2_) influx for photosynthesis and water vapor efflux to prevent leaf desiccation. Stomata open and close through changes in the volume and turgor of the guard cells surrounding the pores. Guard cells transport solutes across the plasma membrane and the tonoplast through highly regulated and interconnected processes that ensure the necessary changes in cell volume and turgor for stomatal movement (Jezek & Blatt, 2017; Horaruang *et al*., 2020; Blatt, 2024).

Transport is also intimately connected with metabolism. The divalent anion malate (Mal) contributes substantially to the osmotic content of guard cells, along with chloride (Cl^−^) and nitrate (NO_3_^−^), serving as counterions for potassium (K^+^). Mal can be imported from the apoplast and sequestered in the vacuole during stomatal opening, with concentrations reaching 100–300 mM in fully open stomata, depending on the species (Martinoia & Rentsch, 1994; Santelia & Lawson, 2016). Import from the apoplast requires ATP to energize the ATP‐binding cassette (ABC) transporter AtABCB14 (Lee *et al*., 2008). Mal can also be produced locally from starch via glycolysis and the anaplerotic pathway (involving phosphoenolpyruvate carboxylase and malate dehydrogenase), from lipid catabolism through acetyl‐CoA entry into the Krebs cycle, and directly within the Krebs cycle itself (Daloso *et al*., 2017).

Starch metabolism plays a central role in regulating stomatal movement (Horrer *et al*., 2016; Santelia & Lunn, 2017; Flütsch *et al*., 2020; Dang *et al*., 2024). Horrer *et al*. (2016) showed that the starch‐degrading enzymes α‐amylase 3 (AMY3) and β‐amylase 1 (BAM1) are essential for initiating starch degradation in guard cells at the start of the day, in a blue light‐dependent manner. In the *amy3bam1* double mutant, guard cells accumulate excessive starch throughout the circadian cycle and fail to show typical degradation patterns, correlating with impaired rapid stomatal opening (Horrer *et al*., 2016). At the plasma membrane, the hexose transporters Sugar Transport Protein 1 and 4 (STP1 and STP4) are critical for coordinating carbohydrate fluxes between mesophyll and guard cells (Flütsch *et al*., 2020). In the *stp1stp4* double mutant, guard cells are devoid of starch, and light‐induced stomatal opening is severely compromised, underscoring the importance of mesophyll‐derived carbohydrates in supporting guard cell metabolism and function (Flütsch *et al*., 2020).

To coordinate stomatal movement with photosynthesis, guard cells must sense and respond to the partial pressure of CO_2_ within the leaf air space (*p*C_*i*_), such that its depletion by photosynthesis promotes stomatal opening (Mott, 1990; Blatt *et al*., 2022). The aerial surfaces of terrestrial plants are largely impermeable to CO_2_, requiring that the gas diffuse through the stomatal pore into the inner airspace before fixation by photosynthesis in the mesophyll. While atmospheric CO_2_ remains relatively stable, *p*C_*i*_ is estimated to vary by 200 μbar and more over the diurnal cycle (Cowan & Farquhar, 1977; Mott, 1990; Blatt *et al*., 2022)—rising above 500 μbar at night due to respiration and dropping rapidly to 150–200 μbar at dawn when photosynthesis begins (Blatt *et al*., 2022). These fluctuations raise important questions about the role of CO_2_ in guard cell metabolism and its integration with membrane transport.

Most research has focused on the effects of elevated CO_2_ on guard cell signaling and stomatal behavior, while the impact of reduced CO_2_ has received far less attention (Mott, 1990; Mott & Peak, 2018). This is a notable knowledge gap, given that bioengineering strategies to enhance photosynthesis – such as introducing C_4_‐like pathways – are likely to reduce *p*C_*i*_, thereby altering guard cell function and organic composition (Ermakova *et al*., 2021; Roell *et al*., 2021; Scheffen *et al*., 2021). Little is known about how CO_2_ influences guard cell metabolism, although elevated CO_2_ has been shown to promote starch synthesis (Azoulay‐Shemer *et al*., 2016). Given the critical role of carbohydrate metabolism in fine‐tuning stomatal behavior, especially during the early light phase (Horrer *et al*., 2016; Flütsch *et al*., 2020), the present study investigates whether low CO_2_ could significantly impact Mal and starch dynamics in guard cells. We demonstrate here that experimentally lowering CO_2_ levels before and during early daylight promotes Mal accumulation, contributing to enhanced stomatal opening. The results indicate that low CO_2_‐induced starch degradation provides the primary carbon skeletons for Mal synthesis, with additional inputs from hexose import via STP1 and STP4. These findings highlight the importance of integrating external CO_2_ cues with internal carbohydrate metabolism to regulate stomatal responses.

## Materials and Methods

### Plant growth

*Arabidopsis thaliana* L. plants were grown under a short‐day 9 : 15 light : dark regime, with 120 μmol m^−2^ s^−1^ PAR, at 22°C : 18°C and relative humidity of 60% : 70%. Experiments were conducted on 7–8 wk‐old plants. The *amy3bam1* and *stp1stp4* mutants were previously described (Horrer *et al*., 2016; Flütsch *et al*., 2020). Seeds were sown in 10 cm pots containing nutrient‐rich Levington F2 + S3 compost (Coulders, Glasgow, UK). After sowing, seeds were stratified at 4°C in darkness for 48 h, then germinated under a plastic lid (> 95% RH) for 2 wk. Plants were subsequently transplanted individually for Mal and gas exchange analysis (Jezek *et al*., 2021; Horaruang *et al*., 2022) and for starch analysis (Flütsch *et al*., 2018).

### Low CO_2_ treatment

For starch phenotyping, 7–8 wk‐old plants were transferred to a SciBrite LED plant growth chamber (Percival Scientific, Perry, GA, USA) with CO_2_ control system, 3 d before experiments for ambient CO_2_ acclimation. Control samples were collected at the end of the night (EON) and at 1 and 2 h into the light period. For low CO_2_ treatment, 100 μbar CO_2_ was applied beginning 30 min before EON.

To accommodate the much larger volume of tissue needed for Mal quantification, plants were loosely sealed in 25 l clear plastic bags in advance of experiments. Before EON, the bags flushed either with air at ambient CO_2_ or with 100 μbar CO_2_ at 2 l min^−1^ to maintain inflation. Samples were collected at EON, 1 h, and 2 h into the photoperiod. Control plants under ambient CO_2_ were processed identically. For gas exchange measurements, control plants were maintained at 400 μbar CO_2_ in the dark for 2 h, before transfer to 100 μbar CO_2_ and the light. In the low CO_2_ treatment, plants were exposed to 100 μbar CO_2_ for 2 h in the dark before transfer to the light under 100 μbar CO_2_.

### Malate quantification

Guard cells were isolated following the blender method of Kruse *et al*. (1989). For each time point, rosettes from 40 plants were blended in ice‐water slurry and filtered through 53 μm nylon mesh. Suspensions were washed and examined by microscope for viability and guard cell purity before counting using a hemocytometer to determine guard cell density. Filtered cells were frozen at −80°C, ground in liquid nitrogen, and heated at 95°C for 15 min. After centrifugation (16 000 ***g***, 10°C, 50 min), Mal was quantified using the l‐Malate Assay Kit (LMAL‐116A; Megazyme, Wicklow, Ireland). Total cell volumes were calculated from the average guard cell size and measured cell densities. Values were normalized to the total cell volume in the sample.

### Starch granule quantification

Starch staining followed (Flütsch *et al*., 2018) with minor changes. Epidermal peels from the 6^th^/7^th^ leaf were fixed in 50% methanol and 10% acetic acid at 4°C for 12 h, decolorized with 80% ethanol (60°C–70°C), and stained with 1% periodic acid followed by sodium metabisulphite (100 mM), 0.15 N HCl, and propidium iodide (0.1 g l^−1^). Peels were mounted in chloral hydrate overnight and fixed with Hoyer's solution. Imaging was performed on a Zeiss LSM 780 confocal microscope. Starch area was quantified from maximum projections using 5 stomata per replicate (4 replicates/treatment/timepoint).

### Gas exchange

Gas exchange was measured using the LI‐6800 system (Li‐Cor, Lincoln, NE, USA) with a 6800‐01A Multiphase Flash Fluorometer. Whole plants were sealed with plastic film to minimize soil gas exchange. Light was provided at 22°C with 10% blue/90% red LED and 60% RH. CO_2_ was held at 400 μbar otherwise noted. At least three plants per genotype were tested. Rosette area was normalized using imagej v.1.51. Stomatal conductance ($g_{s}$) was logged every minute and fitted with exponential function $g_{s}=g_{s0}+a\left(1-e^{-\mathrm{bx}}\right)$. The halftime ($t_{1/2}$) of the response was calculated as $t_{1/2}=\frac{\log_{e}\;2}{b.}$

### OnGuard modeling and statistics

Quantitative modeling was conducted using OnGuard3e (Chen *et al*., 2012; Hills *et al*., 2012; Wang *et al*., 2017; Jezek *et al*., 2021; Nguyen *et al*., 2023). OnGuard3e models used the standard wild‐type Arabidopsis parameter set and were driven through a diurnal light : dark cycle as described previously (Jezek *et al*., 2021; Blatt *et al*., 2022; Horaruang *et al*., 2022; Nguyen *et al*., 2023). Steps in CO_2_ partial pressure and light were imposed on this cycle as indicated, and all model outputs were derived from this cycle. Constant apoplastic solute contents were defined, and primary, energy‐dependent transport, sucrose, and malic acid synthesis within the guard cell were coupled to light as before. Light input also contributed to the rate of carbon fixation by the leaf mesophyll and, hence, to the sink for CO_2_ within the leaf according to established relationships between light, CO_2_, and carbon assimilation (Jezek *et al*., 2021). All other model parameters were fixed. The properties of the individual transporters, metabolism, and buffering reactions thus responded only to changes in model variables arising from the parameters encoded in the model. OnGuard3e and the relevant parameter set for Arabidopsis are freely available for academic users for download from www.psrg.org.uk and www.plantscienceglasgow.org.

As OnGuard outputs are determined by the interactions of the ordinary differential equations that describe each of the underlying processes, statistical analysis of these outputs is meaningless. Statistical significance was assessed by one‐way ANOVA with Dunnett or two‐tailed Student's *t*‐test and reported at *P* < 0.05 unless otherwise noted.

## Results

### Low CO_2_ treatment promotes guard cell starch degradation

**Fig. 1:**
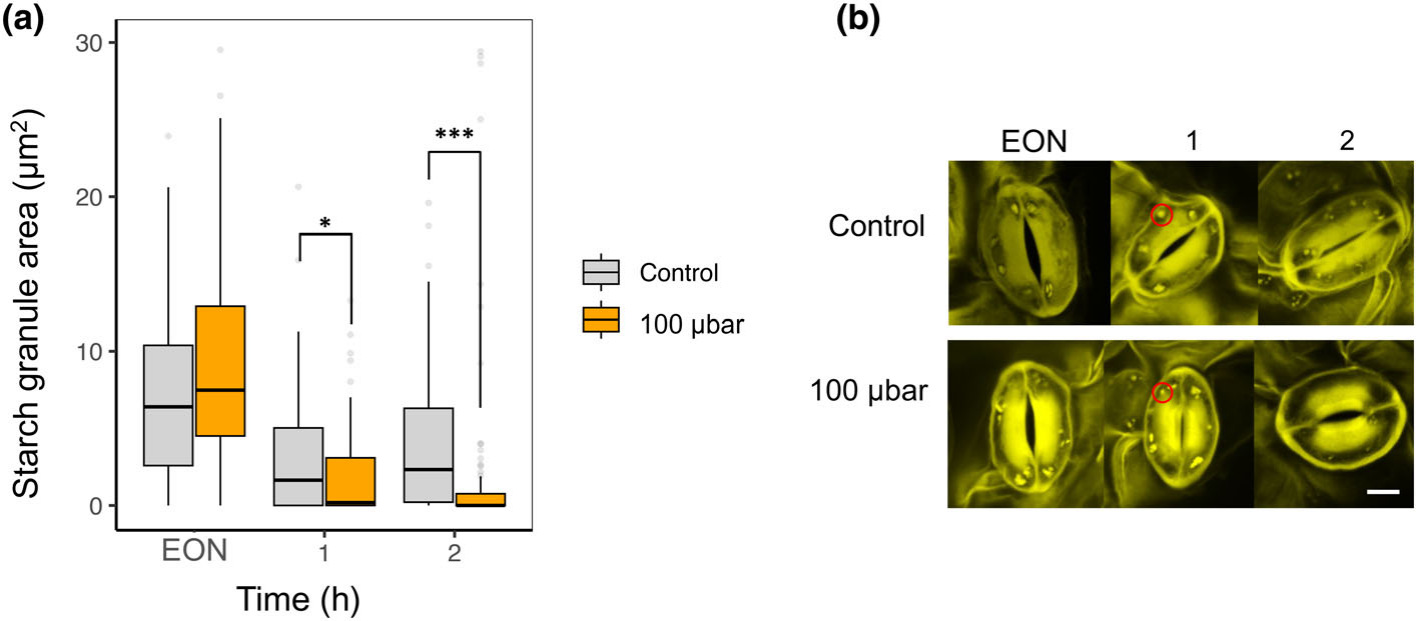
Starch dynamics in *Arabidopsis thaliana* wild‐type guard cells from intact leaves over the first 2 h of the diel cycle under low CO_2_ treatment. (a) Guard cell starch amounts expressed as starch granule area (μm^2^) of control plants (400 μbar, gray) and plants exposed to low CO_2_ (100 μbar, orange). Low CO_2_ treatment was applied 30 min before the start of the day to ensure the desired CO_2_ concentration was achieved at the end of the night (EON). Plants were harvested at EON, 1, and 2 h into the light (150 μmol m^−2^ s^−1^). Data are from three independent experiments with 120 guard cells per treatment per time point from a total of 12 plants. Asterisks indicate significant differences (*, *P* ≤ 0.05; ***, *P* ≤ 0.001) between control and treatment for each time point, after Student's *t*‐test. Error bars: 10^th^ to 90^th^ percentiles. (b) Visualization of starch granules in *A. thaliana* wild‐type guard cells (red circles) of control plants (400 μbar) and low CO_2_ (100 μbar)‐treated plants at the end of the night (EON), and at 1 and 2 h into the light. Bars, 10 μm.

We used propidium iodide staining combined with confocal microscopy to assess the effects of low CO_2_ on guard cell starch metabolism (Horrer *et al*., 2016; Flütsch *et al*., 2018). Plants grown under ambient CO_2_ (400 μbar) were transferred to 100 μbar CO_2_ in the dark 30 min before the onset of the light period, ensuring reduced internal CO_2_ levels at the EON. Leaf samples for guard cell starch staining were collected at the EON, 1 and 2 h into the photoperiod. Starch degradation was initiated upon light transition in both ambient and low CO_2_ conditions, consistent with the diel regulation of guard cell starch turnover. However, plants exposed to 100 μbar CO_2_ showed lower guard cell starch content after just 1 h of light exposure compared to those maintained at ambient CO_2_ (Fig. 1a). After 2 h into the light, guard cells of control plants began to re‐accumulate starch as expected, while starch remained low in plants kept at low CO_2_ concentrations. Confocal imaging revealed smaller and sparser starch granules under low CO_2_, suggesting that CO_2_ availability modulates the diurnal pattern of starch metabolism in guard cells (Fig. 1b).

### Malate accumulation is observed during low CO_2_ treatment

**Fig. 2:**
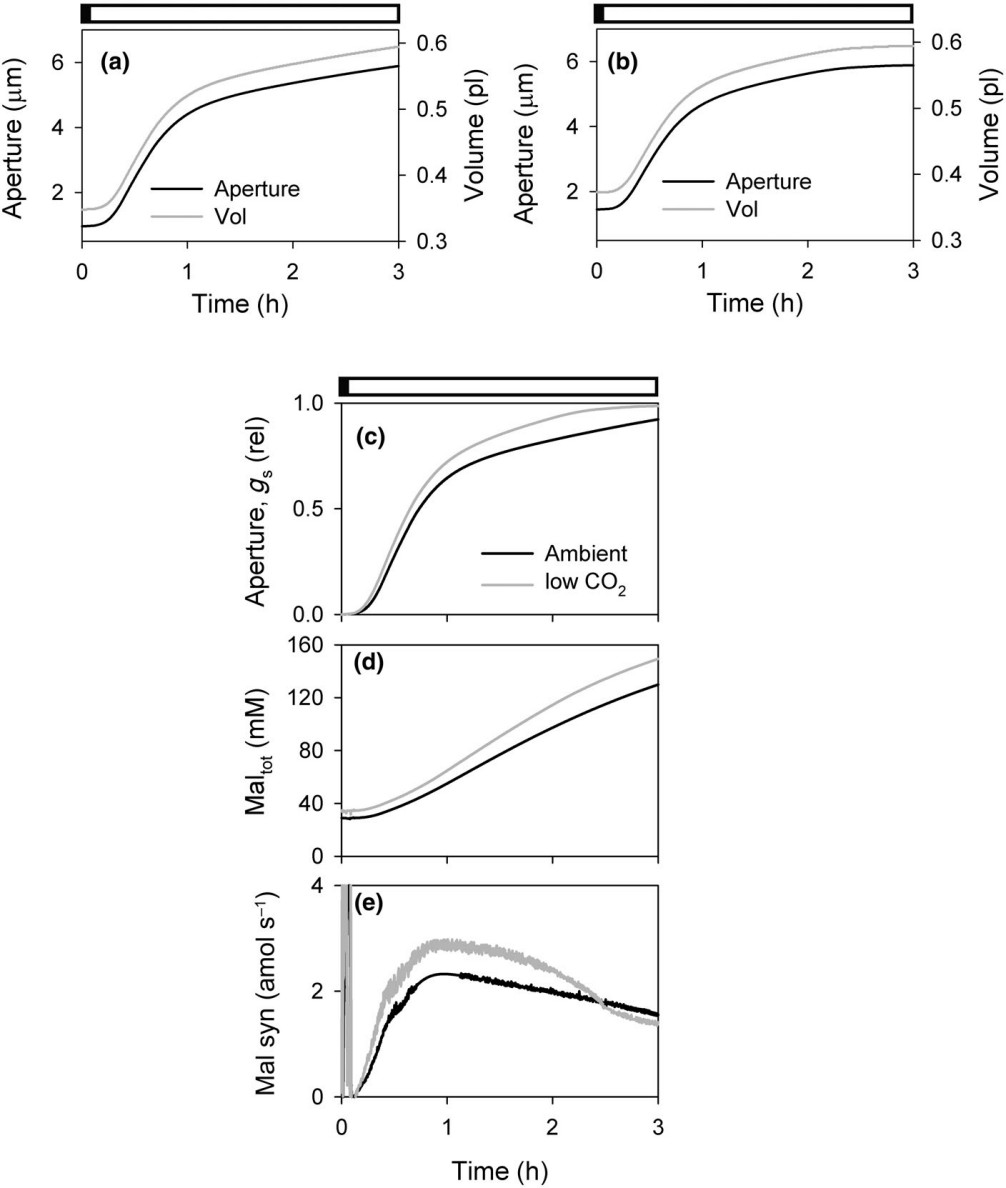
Outputs from OnGuard3e simulations of stomatal dynamics. Simulation outputs generated using the standard Arabidopsis wild‐type parameter set (see Horaruang *et al*., 2022; Nguyen *et al*., 2023) under ambient (400 μbar) and 100 μbar CO_2_ over the first 3 h of daylight. For low CO_2_, atmospheric CO_2_ partial pressure was reduced to 100 μbar 30 min before the start of the daylight period (0 h). Bars (above) indicate the dark to light transition. Selected outputs are of (a) stomatal aperture and guard cell volume under ambient carbondioxide (CO_2_); (b) Same as in (a), but with 100 μbar CO_2_. (c) Relative aperture/stomatal conductance scaled to the diel dynamic range. (d) Total Mal concentration; and (e) Mal synthesis rate. Note that ambient and low CO_2_ outputs are overlaid in (c–e) for ease of comparison. The full set of relevant simulation outputs is included in Supporting Information Fig. S1.

Mal directly contributes to the osmotic potential required for stomatal opening. However, its diel dynamics under varying CO_2_ conditions remain poorly characterized (Talbott & Zeiger, 1993; Asai *et al*., 2000). To this end, the OnGuard3e modeling platform (Chen *et al*., 2012; Hills *et al*., 2012; Wang *et al*., 2017; Jezek *et al*., 2021; Nguyen *et al*., 2023) was used to simulate the effects of low CO_2_ on stomatal dynamics and Mal content. Simulations followed a standard diurnal light regime, with CO_2_ levels reduced to 100 μbar before light onset. Modeling showed an increase in stomatal aperture in the dark (Fig. 2a: control; Fig. 2b: low CO_2_; see also Supporting Information Fig. S1), in agreement with previous studies reporting increased stomatal aperture in darkness under low CO_2_ conditions (Morison & Jarvis, 1983; Olsen *et al*., 2002; Doi & Shimazaki, 2008). These findings further support the concept of a functional interplay between light and CO_2_ signaling in the regulation of stomatal behavior (Blatt *et al*., 2022).

**Fig. 3:**
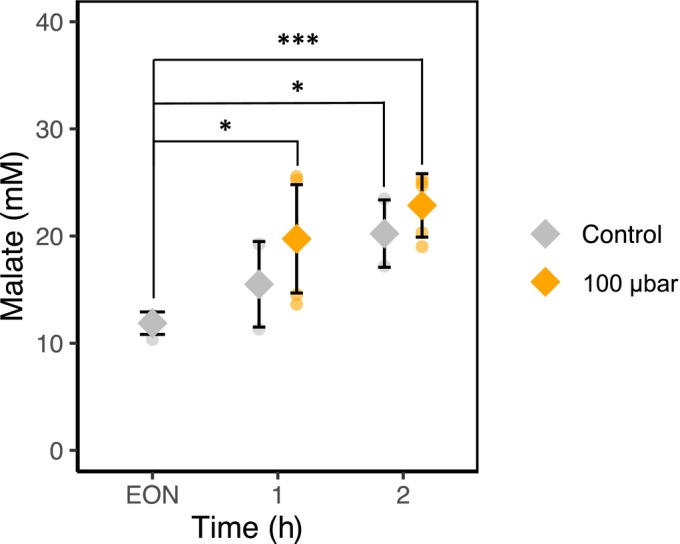
Malate concentration of wild‐type *Arabidopsis thaliana* guard cells under 400 μbar carbondioxide (CO_2_) (gray symbols) and 100 μbar CO_2_ (orange symbols). Samples were collected at the end of night (EON), and after 1 and 2 h of light. Each data point represents the mean of at least three biological replicates, each replicate comprising guard cells from 30 to 35 g of 8‐wk‐old plants. Error bars are ±SD. Asterisks mark statistically significant differences between EON and the subsequent time points (*, *P* ≤ 0.05; ***, *P* ≤ 0.001) based on *post‐hoc* Dunnett test.

OnGuard3e simulations also predicted an accelerated stomatal opening, accompanied by a counter‐intuitive increase in Mal content and elevated Mal synthesis under low CO_2_ conditions (Figs 2c–e, S1). To experimentally validate these predictions, Mal levels were measured in guard cell‐enriched leaf tissues of wild‐type *A. thaliana* at the EON and after 1 and 2 h of light exposure. Plants were divided into two groups: one maintained at ambient 400 μbar CO_2_ and the other transferred to 100 μbar CO_2_ 30 min before EON. Under ambient CO_2_ conditions, Mal levels increased gradually throughout the early photoperiod, although no statistically significant differences were observed compared to EON (Fig. 3). By contrast, plants exposed to low CO_2_ exhibited a rapid and significant rise in Mal content as early as 1 h after light onset, which persisted at 2 h, indicating a strong metabolic response to reduced CO_2_ availability (Fig. 3).

### Starch breakdown and hexose uptake contributes to Mal accumulation under low CO_2_

**Fig. 4:**
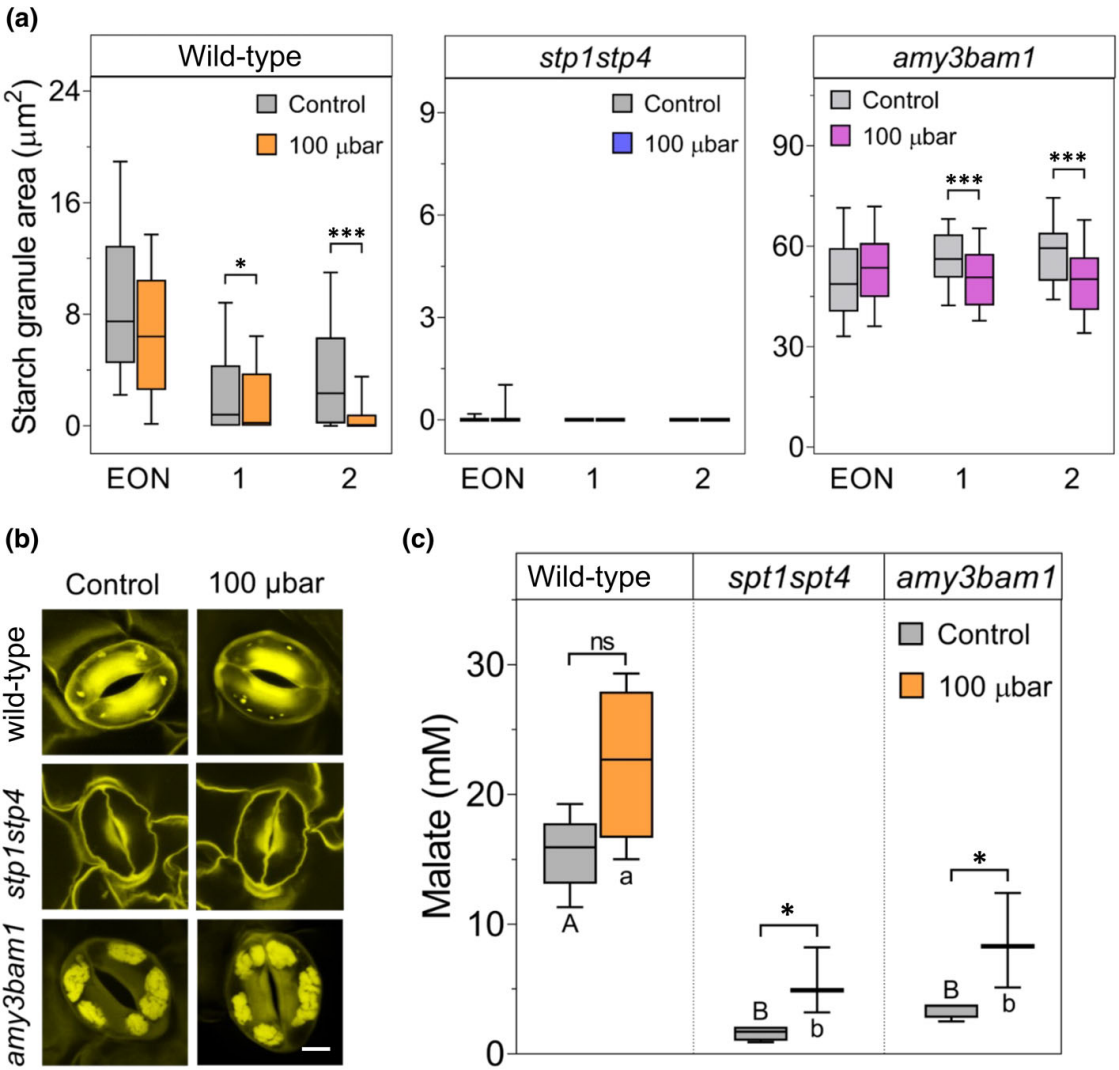
Impact of metabolic and transport mutations on guard cell starch and malate. (a) Guard cell starch content expressed as starch granule area (μm^2^) in wild‐type *Arabidopsis thaliana* and the mutants *stp1stp4* and *amy3bam1* at EON and after 1 and 2 h of 100 μbar CO_2_ treatment compared to the control under ambient (400 μbar) CO_2_. Asterisks indicate significant differences (*, *P* ≤ 0.05; ***, *P* ≤ 0.001) between control and treatment at each time point by *post‐hoc* Student *t*‐test. Error bars represent the 10^th^ to 90^th^ percentiles. Note the different *y*‐axis scales. The number of guard cells per genotype per time point and treatment was 120 in wild‐type, 40 in *stp1stp4*, and 80 in *amy3bam1*. (b) Visualization of starch granules in guard cells of wild‐type *A. thaliana* and the mutants *stp1stp4* and *amy3bam1* plants under control conditions and after 1 h 100 μbar CO_2_ treatment. Bars, 10 μm. (c) Guard cell malate content for wild‐type *A. thaliana* and the *stp1stp4* and *amy3bam1* mutants at 1 h of 100 μbar CO_2_ treatment compared to the control under ambient (400 μbar) CO_2_. Each data point represents the average of at least three biological replicates as in Fig. 3. Error bars represent the 10^th^ to 90^th^ percentiles. Letters denote significant differences between genotypes within the same treatment, whereas asterisks indicate significant differences between treatments within the same genotype after *post‐hoc* Student *t*‐test.

To examine the contribution of starch turnover and hexose availability to Mal accumulation under low CO_2_, two double mutants were analyzed: *amy3bam1*, defective in starch degradation, and *stp1stp4*, deficient in hexose import, and devoid of guard cell starch as a consequence of reduced substrate availability. Both mutants were previously shown to exhibit reduced stomatal opening due to metabolic limitations (Horrer *et al*., 2016; Flütsch *et al*., 2020). As expected, *stp1stp4* mutants lacked detectable starch granules in guard cells under both ambient and low CO_2_ (Fig. 4a,b). By contrast, *amy3bam1* retained high starch levels across all time points and conditions, with only a modest reduction in starch content at 1 and 2 h after dawn. This decrease was significantly smaller compared to that observed in wild‐type guard cells, confirming impaired starch degradation. Under control conditions, *amy3bam1* and *stp1stp4* mutants had significantly lower Mal compared to the wild‐type (Fig. 4c). In response to low CO_2_ treatment, both mutants accumulated Mal but not as much as wild‐type (Fig. 4c). In *amy3bam1*, lower Mal accumulation was consistent with limited starch turnover, reinforcing the hypothesis that starch‐derived carbon skeletons are essential for early‐day Mal synthesis. In the *stp1stp4* mutants, Mal accumulation under low CO_2_ could potentially result from alternative carbon sources such as lipid β‐oxidation, Krebs cycle intermediates, or mesophyll‐derived Mal import via the apoplast (Lee *et al*., 2008; Daloso *et al*., 2017). Hexose uptake might sustain Mal synthesis – either directly via glucose (Glc)‐to‐Mal conversion or indirectly by contributing to making starch, which is then later degraded again. Together, these results demonstrate that both starch degradation and apoplastic sugar uptake are essential for robust Mal production during the early photoperiod and contribute to the guard cell response under low CO_2_ conditions.

### Low CO_2_ pretreatment primes fast stomatal opening through starch breakdown and hexose uptake

**Fig. 5:**
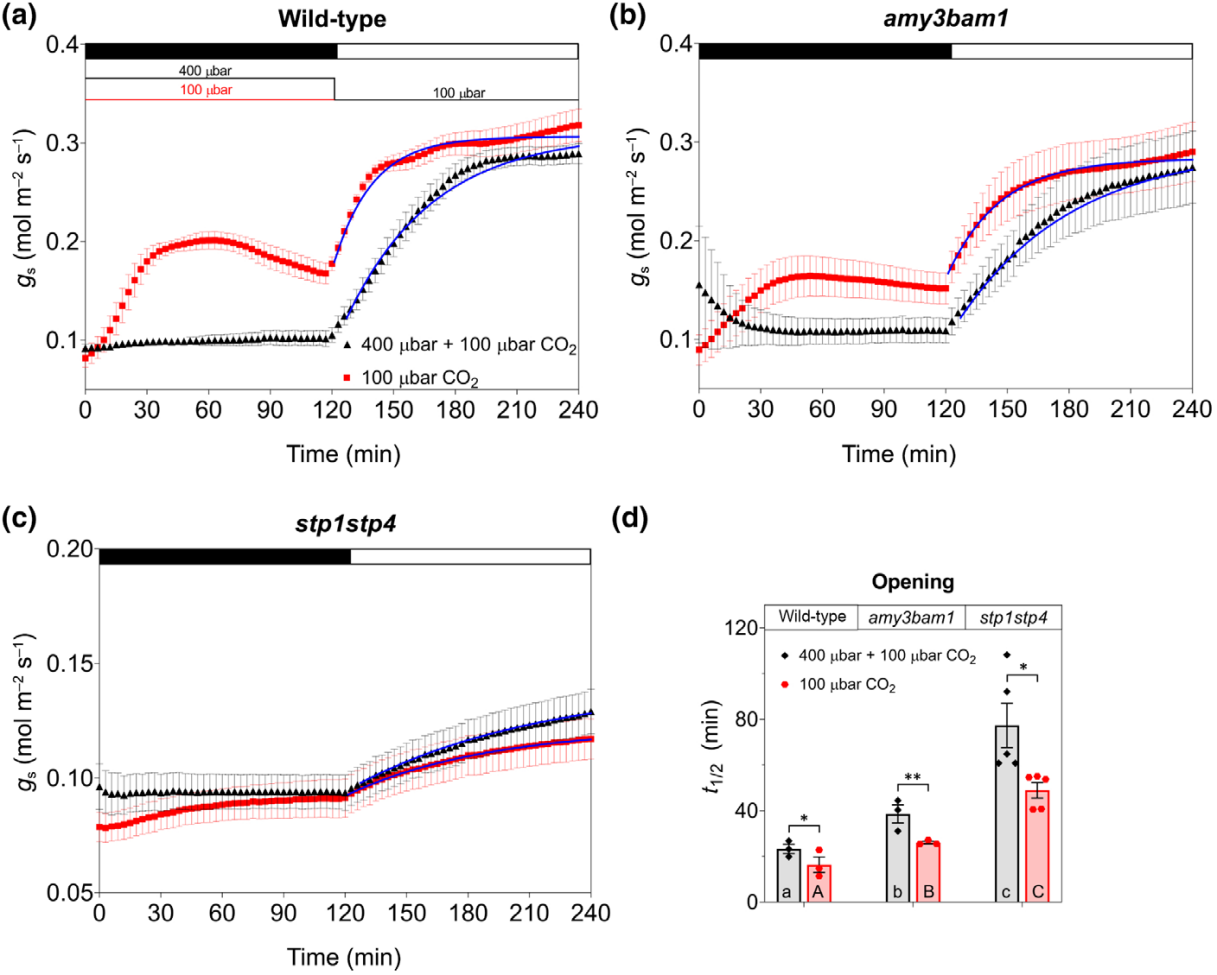
Low carbondioxide (CO_2_) pretreatment primes stomata for faster opening. Stomatal conductance (*g*_s_) measurements from (a) wild‐type *Arabidopsis thaliana* (*n* = 3 and 4), and the mutants (b) *amy3bam1* (*n* = 3 and 7), and (c) *stp1stp4* (*n* = 5 and 6), under control and extended 100 μbar CO_2_ treatments, respectively. Control experiments (400 μbar → 100 μbar) entailed concurrent transfer from 400 μbar CO_2_ and dark to 100 μbar CO_2_ and light. Extended 100 μbar CO_2_ treatment entailed transfer to low CO_2_ 2 h before the start of daylight. Dark to light and CO_2_ transitions are indicated (color‐coded, above). (d) Halftimes (*t*_1/2_) for stomatal opening derived from first‐order exponential fits (a–c, blue solid lines). Bars are means ± SE with independent experimental data shown as points. Letters denote significant differences between genotypes under the same treatment, whereas asterisks indicate significant differences between treatments for the same genotype after *post‐hoc* Student *t*‐test (*, *P* ≤ 0.05; **, *P* ≤ 0.01).

Having established that starch degradation and hexose uptake both contribute to Mal accumulation under low CO_2_, we asked whether these metabolic changes influenced stomatal kinetics. Gas exchange measurements were conducted to assess stomatal conductance (*g*_s_) in wild‐type, *amy3bam1*, and *stp1stp4* plants during a transition from dark to light (200 μmol m^−2^ s^−1^ PAR), with or without a 2‐h low CO_2_ pretreatment in darkness (Fig. 5a–c). Under control condition, plants were maintained at 400 μbar CO_2_ in darkness, followed by exposure to 100 μbar CO_2_ upon illumination.

In wild‐type plants, low CO_2_ pretreatment led to a significant increase in *g*_s_ even before the light onset (Fig. 5a). This enhancement is consistent with earlier reports showing that low CO_2_ alone can promote partial stomatal opening in the dark (Morison & Jarvis, 1983; Olsen *et al*., 2002; Doi & Shimazaki, 2008), and it was also predicted by the OnGuard3e simulations (Fig. 2a,b). Upon illumination, pretreated plants also exhibited a steeper rate of increase in *g*_s_ compared to nonpretreated controls, resulting in significantly shorter halftimes to reach maximal aperture (Fig. 5d). The elevated initial *g*_s_ also reduced the differrential to the steady‐state plateau, a parameter previously correlated with stomatal opening speed (Wachendorf & Küppers, 2017).

The *amy3bam1* mutant also benefited from the low CO_2_ pretreatment, showing partial stomatal preopening in darkness and a more modest acceleration in stomatal opening kinetics to light (Fig. 5b). However, both the initial *g*_s_ values and the half time of opening under low CO_2_ were lower compared to that of wild‐type (Fig. 5d). Starch degradation likely contributed to establishing the initial *g*_s_, in turn influencing the speed and extent of stomatal opening. In *amy3bam1*, reduced initial *g*_s_ under low CO_2_ was associated with slower opening kinetics and lower amplitude, suggesting that impaired starch breakdown limits both the onset and magnitude of the response to light.

Interestingly, the *stp1stp4* mutant failed to show increase in initial *g*_s_ following low CO_2_ pretreatment and exhibited severely impaired stomatal responses overall (Fig. 5c). Both the amplitude and rate of *g*_s_ increase were substantially reduced compared to wild‐type and *amy3bam1*. While a minor reduction in halftime was noted in *stp1stp4* after pretreatment (Fig. 5d), its limited dynamic *g*_s_ range and low overall conductance diminished the physiological relevance of this effect. Nonetheless, the *stp1stp4* phenotype underscores the essential role of sugar import in contributing to Mal synthesis and osmotic adjustment during stomatal opening. In summary, pretreatment with low CO_2_ primes guard cells for faster stomatal opening in response to light, likely through early metabolic shifts that promote Mal synthesis. Although both *amy3bam1* and *stp1stp4* mutants still showed increased Mal levels, their attenuated stomatal responses highlight that starch degradation and sugar uptake contribute not just to Mal production but also to the efficiency and extent of osmotic regulation during both dark and light phases. These findings show that low CO_2_ exposure before illumination activates starch degradation–and potentially sugar import–promoting sugar accumulation for Mal synthesis before light onset. This metabolic priming supports the osmotic changes required for full stomatal opening in the light.

## Discussion

### Deconstructing the unforeseen consequences of low CO_2_

Much of the research on stomatal physiology and photosynthesis has centered on the effects of elevated atmospheric CO_2_, a well‐established consequence of industrialization, and on strategies to improve CO_2_ fixation and water‐use efficiency (WUE = CO_2_ fixed per unit water loss, *A*/*g*_s_) in crops (Lawson & Blatt, 2014; Ort *et al*., 2015; Matthews & Lawson, 2019). Among bioengineering advances, manipulating guard cell membrane transport has shown considerable promise for enhancing both carbon assimilation and WUE (Papanatsiou *et al*., 2019; Horaruang *et al*., 2022). However, such improvements may inadvertently lower the time‐averaged *p*C_*i*_ within the leaf airspace (Blatt *et al*., 2022), raising key questions about how reduced *p*C_*i*_ affects guard cell function. Although the consequences of low *p*C_*i*_ are not yet fully understood, it is well known that guard cell membrane transport is tightly linked to starch and Mal metabolism (Horrer *et al*., 2016). Elevated *p*C_*i*_ is associated with suppressed ion transport and increased sugar and starch accumulation in guard cells (Assmann & Jegla, 2016; Azoulay‐Shemer *et al*., 2016; Santelia & Lawson, 2016; Hiyama *et al*., 2017; Zhang *et al*., 2018; Jezek *et al*., 2021; Blatt, 2024). However, it does not necessarily follow that lowering *p*C_*i*_ produces the inverse effect.

To address this gap, we examined the short‐term effects of low CO_2_ exposure during the early light period (EON–2 h), thereby avoiding confounding factors from long‐term carbon starvation. OnGuard model simulations predicted an unexpected increase in guard cell Mal content and enhanced stomatal opening under reduced atmospheric CO_2_. Our empirical results confirmed these predictions: reducing atmospheric CO_2_ to 100 μbar before light onset significantly increased Mal levels in guard cells and accelerated stomatal opening. Mutant analyses using *amy3bam1* and *stp1stp4* lines indicated that both starch degradation and sugar import contribute to this enhanced Mal synthesis, revealing a previously overlooked aspect of metabolic reprogramming in guard cells in response to low CO_2_.

### Mechanistic insights from OnGuard modeling

The OnGuard platform links low CO_2_ to changes in vacuolar and cytosolic transport. Specifically, Mal flux across the tonoplast is regulated by two main transporters, VMal and VCl, the properties of which have been well‐characterized experimentally (Chen *et al*., 2012; Hills *et al*., 2012; Jezek & Blatt, 2017; Wang *et al*., 2017; Jezek *et al*., 2021). These transporters are both voltage‐sensitive and pH‐dependent, enabling vacuolar Mal accumulation under acidic conditions. Vacuolar acidification relies on the activity of the tonoplast‐localized V‐H^+^‐ATPase and V‐H^+^‐PPase (VH‐ATPase and VH‐PPase), with the latter being inhibited by cytosolic calcium (Ca^2+^) near 100 nM (Kriegel *et al*., 2015). Low atmospheric CO_2_ leads to a drop in *p*C_*i*_ and is predicted to lower cytosolic Ca^2+^ concentration via enhanced Ca^2+^ sequestration into the vacuole by endomembrane Ca^2+^‐ATPases (VCa‐ATPase) (Jezek *et al*., 2021). The reduction in cytosolic‐free [Ca^2+^] enhances both Mal synthesis and its transport into the guard cell vacuole, processes independent of the mesophyll. In turn, Mal accumulation indirectly promotes K^+^ uptake for charge balance and accelerates turgor generation, stomatal opening and conductance. While the connections between these processes can be traced through an analysis of the model response, the overall effect of accumulating carbon skeletons runs counter to intuitive expectation that Mal accumulation should decline when the source of carbon is reduced. While not all model predictions are easily testable, the predicted increase in Mal content was confirmed experimentally (Fig. 3), validating the conclusion that Mal accumulation is both rapid and robust under low CO_2_, either through enhanced synthesis or import.

### A metabolic challenge for stomatal bioengineering

These insights arising from OnGuard modeling and the experiments above have significant implications for stomatal bioengineering. Although enhancing membrane transport in guard cells can improve photosynthetic performance and WUE under fluctuating conditions, it may also disrupt *p*C_*i*_ homeostasis and metabolic balance in guard cells (Blatt *et al*., 2022). Our findings show that even transient reductions in *p*C_*i*_ can rewire guard cell metabolism towards enhanced Mal accumulation, involving both signaling pathways and core carbon metabolism. The dependence on sugar import (via STP1/STP4) and starch degradation (via AMY3/BAM1) underscores the necessity of carbohydrate flux during early light transitions. Both *amy3bam1* and *stp1stp4* mutants showed impaired Mal accumulation under low CO_2_ after 2 h of light. However, gas exchange measurements revealed more complex dynamics: under low CO_2_ pretreatment in darkness, wild‐type plants displayed higher initial *g*_s_ and faster light‐induced opening than *amy3bam1* (Fig. 5), indicating that starch degradation contributes both to the initial opening phase and to predawn metabolic priming, as recently highlighted by Zhang *et al*. (2025). These observations align with prior findings showing that initial stomatal conductance plays a pivotal role in rapid photosynthetic induction under fluctuating light by facilitating immediate CO_2_ uptake and potentially faster Rubisco activation (Kaiser *et al*., 2017; Kang *et al*., 2024). By contrast, *stp1stp4* mutants showed a near‐complete lack of response to low CO_2_ in both pretreatment and light phases (Fig. 5), highlighting the critical role of glucose import – not only for starch synthesis but also for Mal production. Interestingly, *amy3bam1* still exhibited starch degradation under low CO_2_, possibly due to alternative amylase activity; however, this was insufficient to achieve wild‐type Mal levels or conductance (Figs 4, 5). Together, these results establish low CO_2_ not only as a stomatal signal but also as a metabolic cue triggering alternative carbon utilization pathways. Prelight exposure to low CO_2_ primes guard cells for more efficient opening, provided sufficient carbon reserves or imported sugars are available. These findings argue for a more integrative approach to stomatal bioengineering that incorporates both signaling and metabolic dimensions. Such strategies could help create crops with more responsive and efficient stomatal regulation in the face of increasing environmental variability and CO_2_ fluctuations.

## Competing interests

None declared.

## Author contributions

MRB and FALS‐A conceived the project; LP generated the starch data. FALS‐A produced the Mal and stomatal conductance data; MRB ran the OnGuard3 model; LP, FALS‐A, MRB, and DS contributed to the experimental design and manuscript writing. FALS‐A and LP contributed equally to this work.

## Disclaimer

The New Phytologist Foundation remains neutral with regard to jurisdictional claims in maps and in any institutional affiliations.

## Supporting information


**Fig. S1** OnGuard3e simulation of stomatal dynamics and malate metabolism under low CO_2_ conditions using the standard Arabidopsis wild‐type parameter set.
Please note: Wiley is not responsible for the content or functionality of any Supporting Information supplied by the authors. Any queries (other than missing material) should be directed to the *New Phytologist* Central Office.

## Data Availability

Data generated in this study are included in the article and its Fig. S1.
