# Supplementary material for: Guard cell starch and malate metabolism facilitate stomatal opening in response to low CO2
Source: New Phytol. 2025 Oct 11;249(1):189–97. doi: 10.1111/nph.70639 (PMC12676080; doi:10.1111/nph.70639)
Supplement: Supplementary file 1 — Fig. S1 OnGuard3e simulation of stomatal dynamics and malate metabolism under low CO2 conditions using the standard Arabidopsis wild‐type parameter set. Please note: Wiley is not responsible for the content or functionality of any Supporting Information supplied by the authors. Any queries (other than missing material) should be directed to the New Phytologist Central Office. [file NPH-249-189-s001.pdf]

New Phytologist Supporting Information

Guard cell Starch and Malate Metabolism Facilitate Stomatal Opening in Response to Low CO_2

Fernanda A. L. Silva-Alvim, Lucia Piro, Diana Santelia, Michael R. Blatt

Accepted 16. September 2025

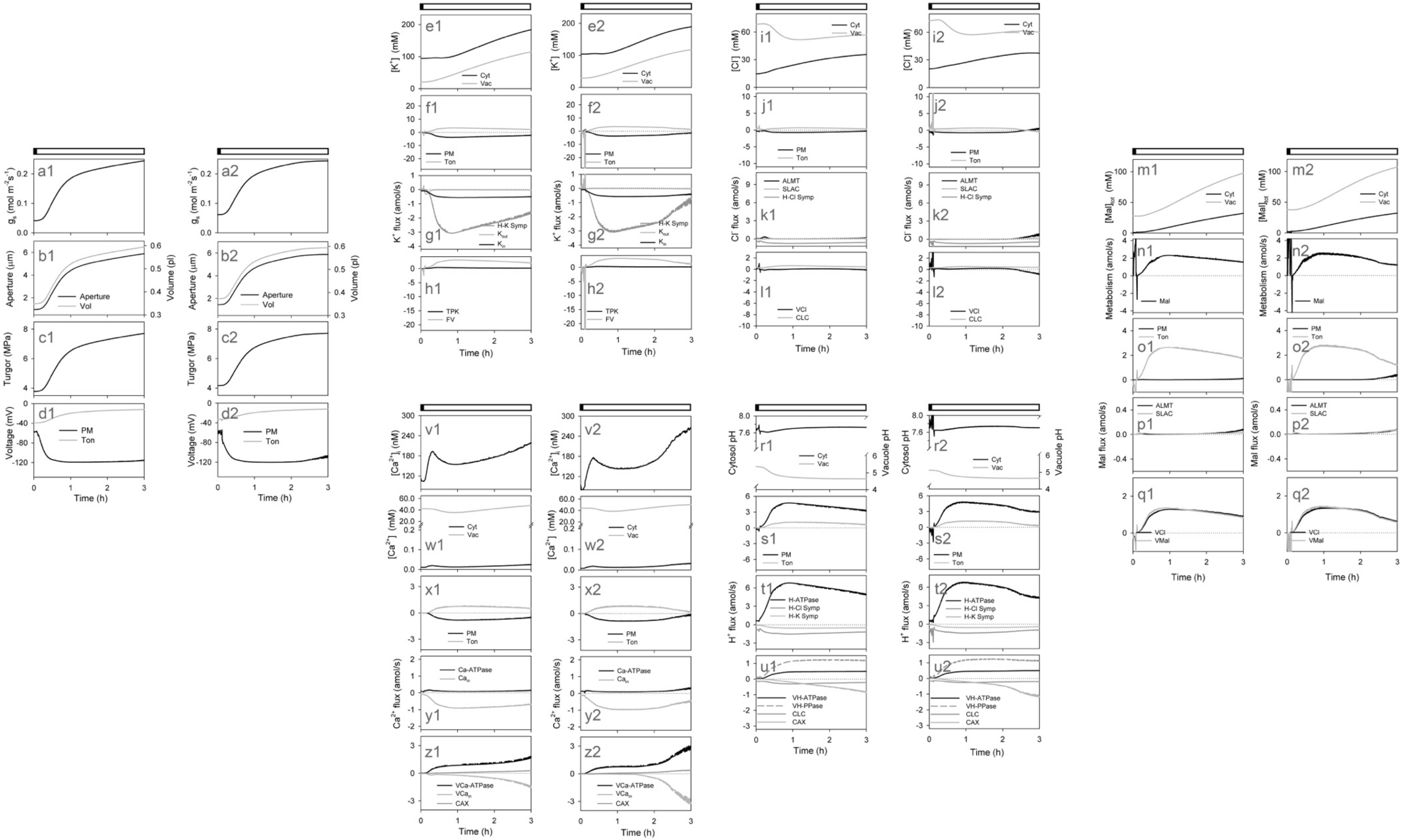

a1
a2
b1
b2
c1
c2
d1
d2
Aperture
Vol
Aperture (μm)
Volume (pl)
Turgor (MPa)
Voltage (mV)
PM
Ton
Time (h)
e1
e2
f1
f2
g1
g2
h1
h2
Cyt
Vac
H-K Symp
TPK
FV
K+ flux (amol/s)
i1
i2
j1
j2
k1
k2
l1
l2
ALMT
SLAC
H-Cl Symp
VCl
CLC
Cl- flux (amol/s)
m1
m2
n1
n2
o1
o2
p1
p2
q1
q2
Mal
VMal
Metabolism (amol/s)
Mal flux (amol/s)
v1
v2
w1
w2
x1
x2
y1
y2
z1
z2
Ca-ATPase
VCa-ATPase
CAX
Ca2+ flux (amol/s)
r1
r2
s1
s2
t1
t2
u1
u2
Cytosol pH
Vacuole pH
H-ATPase
VH-ATPase
VH-PPase
H+ flux (amol/s)

Supplementary Figure S1. OnGuard3e simulation of stomatal dynamics and Mal metabolism under low CO_2 conditions using the standard Arabidopsis wild-type parameter set (see Horaruang et al 2022; Nguyen et al 2023). Bars (above) indicate transition from dark to light. Panels a1-z1 are simulation outputs with 400 μbar CO_2; panels a2-z2 are simulation outputs with 100 μbar CO_2 introduced beginning 30 min before end of night (EON) at 0 h. Fluxes are indicated relative to the cytosolic compartment so that positive flux is movement out of the cytosol. Full descriptions of the transporters and their physiological characteristics will be found in Hills et al 2012 and Jezek and Blatt 2017. Outputs are grouped as:

(a) Stomatal conductance (g_s); (b) stomatal aperture and guard cell volume; (c) guard cell turgor; and (d) plasma membrane and tonoplast voltage;

(e) guard cell [K^+] in the cytoplasm (Cyt) and vacuole (Vac); (f) total K^+ flux across the plasma membrane (PM) and tonoplast (Ton); (g) plasma membrane flux through H^+-K^+ symport (H-K Symp), GORK (K_{out}) and KAT (K_{in}) channels; and (h) tonoplast K^+ flux through TPK- and FV-type channels;

(i) guard cell [Cl^-] in the cytoplasm (Cyt) and vacuole (Vac); (j) total Cl^- flux across the plasma membrane (PM) and tonoplast (Ton); (k) plasma membrane flux through H^+-Cl^- symport (H-Cl Symp), R-type (ALMT) and S-type (SLAC) channels; and (l) tonoplast Cl^- flux through H^+-Cl^- symport (CLC) and VCL-type channels;

(m) total guard cell [Mal] in the cytoplasm (Cyt) and vacuole (Vac); (n) Mal metabolism (synthesis, positive; breakdown, negative); (o) total Mal flux across the plasma membrane (PM) and tonoplast (Ton); (p) plasma membrane flux through R-type (ALMT) and S-type (SLAC) channels; and (q) tonoplast Mal flux through and VCL-type and VMal channels;

(r) guard cell pH in the cytoplasm (Cyt) and vacuole (Vac); (s) total H^+ flux across the plasma membrane (PM) and tonoplast (Ton); (t) plasma membrane H^+ flux through H^+-Cl^- symport (H-Cl Symp), H^+-K^+ symport (H-K Symp), and H^+-ATPases (H-ATPase); and (u) tonoplast H^+ flux through H^+-Cl^- symport (CLC), H^+-Ca^{2+} antiport (CAX), VH^+-ATPases (VH-ATPase) and VH^+-PPases (VH-PPase);

(v) guard cell cytosolic-free [Ca^{2+}] ([Ca^{2+}]$_i$); (w) total [Ca^{2+}] in the cytoplasm (Cyt) and vacuole (Vac); (x) total Ca^{2+} flux across the plasma membrane (PM) and tonoplast (Ton); (y) plasma membrane flux through Ca^{2+}-ATPases (Ca-ATPase) and Ca^{2+} channels (Ca_{in}); and (z) tonoplast Ca^{2+} flux through VCa^{2+}-ATPases (VCa-ATPase), Ca^{2+} channels (VCa_{in}), and H^+-Ca^{2+} antiport (CAX).

Citations

Hills A, Chen ZH, Amtmann A, Blatt MR, Lew VL. 2012. OnGuard, a Computational Platform for Quantitative Kinetic Modeling of Guard cell Physiology. *Plant Physiology* **159**: 1026–1042.

Horaruang W, Klejchová M, Carroll W, Silva-Alvim F, Waghmare S, Papanatsiou M, Amtmann A, Hills A, Alvim J, Blatt MR, *et al.* 2022. Engineering a K^+ channel 'sensory antenna' enhances stomatal kinetics, water use efficiency and photosynthesis. *Nature Plants* **8**: 1262–1274.

Jezek M, Blatt MR. **2017**. The Membrane Transport System of the Guard cell and Its Integration for Stomatal Dynamics. *Plant Physiology* **174**: 487–519.

Nguyen T-H, Silva-Alvim, FAL, Hills, A, Blatt, MR. 2023. OnGuard3e: a predictive, ecophysiology-ready tool for gas exchange and photosynthesis research. *Plant, Cell and Environment* **46**:3644-3658.
